# Regulatory Effects of Sestrin 3 (SESN3) in BCR-ABL Expressing Cells

**DOI:** 10.1371/journal.pone.0078780

**Published:** 2013-11-18

**Authors:** Eliza Vakana, Ahmet Dirim Arslan, Amy Szilard, Jessica K. Altman, Leonidas C. Platanias

**Affiliations:** 1 Robert H. Lurie Comprehensive Cancer Center and Division of Hematology-Oncology, Northwestern University Medical School, Chicago, Illinois, United States of America; 2 Division of Hematology-Oncology, Jesse Brown Veterans Affairs Medical Center, Chicago, Illinois, United States of America; Emory University, United States of America

## Abstract

Chronic myeloid leukemia (CML) and Ph+ acute lymphoblastic leukemia (ALL) are characterized by the presence of the BCR-ABL oncoprotein, which leads to activation of a plethora of pro-mitogenic and pro-survival pathways, including the mTOR signaling cascade. We provide evidence that in BCR-ABL expressing cells, treatment with tyrosine kinase inhibitors (TKIs) results in upregulation of mRNA levels and protein expression of sestrin3 (SESN3), a unique cellular inhibitor of mTOR complex 1 (mTORC1). Such upregulation appears to be mediated by regulatory effects on mTOR, as catalytic inhibition of the mTOR kinase also induces SESN3. Catalytic mTOR inhibition also results in upregulation of SESN3 expression in cells harboring the TKI-insensitive T315I-BCR-ABL mutant, which is resistant to imatinib mesylate. Overexpression of SESN3 results in inhibitory effects on different Ph+ leukemic cell lines including KT-1-derived leukemic precursors, indicating that SESN3 mediates anti-leukemic responses in Ph+ cells. Altogether, our findings suggest the existence of a novel mechanism for the generation of antileukemic responses in CML cells, involving upregulation of SESN3 expression.

## Introduction

Chronic myeloid leukemia (CML) and Ph+ acute lymphoblastic leukemia (Ph+ ALL) are characterized by the presence of the BCR-ABL oncoprotein, the product of the Philadelphia chromosome (Ph) resulting from the aberrant translocation between chromosomes 9 and 22 [1], [2]. BCR-ABL exhibits constitutively active tyrosine kinase activity, leading to the engagement of many anti-apoptotic and pro-proliferative effector cascades [3], [4], [5], [6], [7], [8], [9]. Specific inhibition of BCR-ABL by first- and second-generation tyrosine kinase inhibitors (TKIs) targeting the kinase domain of BCR-ABL, such as imatinib, nilotinib and dasatinib, has revolutionized the treatment of Ph+ malignancies [10], [11]. Despite the potent effects of second-generation BCR-ABL inhibitors, a subset of patients still harbor certain BCR-ABL mutations, such as the T315I mutation, which is refractory to first- and second-generation TKIs *in vitro* and *in vivo* [12], [13], [14]. More extensive identification of patient-specific BCR-ABL mutations and realization of emerging resistance to second-generation TKIs led to rigorous efforts in designing and developing new specific inhibitors that can block the activity of the T315I BCR-ABL mutant. One such agent is the third-generation TKI, ponatinib, as initial studies of ponatinib in CML patients indicated that it has clinical activity with remarkable hematologic and cytogenetic responses even in patients with the BCR-ABL-T315I mutation [15], [16]. Despite the success of ponatinib and its recent approval for the treatment of TKI resistant patients, a new study shed light on three newly identified BCR-ABL mutations, L248R, T315V and F317R, detected in CML and Ph+ ALL patients that appear to exhibit differential resistance to TKIs, including the third-generation inhibitors ponatinib and DCC-2036, underscoring the need for novel therapies for resistant Ph+ CML and ALL [17].

While selective targeting of BCR-ABL with new agents may be an effective approach to overcome resistance associated with BCR-ABL mutations, cellular resistance has been documented with mutations unrelated to the BCR-ABL oncoprotein, such as overexpression of the Lyn tyrosine kinase, leading to imatinib resistance by Bcl-2 activation [18], [19], [20]. A potential strategy to counteract the various modes of resistance could involve targeting signaling cascades that mediate diverse cellular signals, providing an important and possibly more effective approach to reverse leukemic cell resistance in BCR-ABL malignancies.

An important mitogenic pathway engaged by BCR-ABL and readily studied as an alternative therapeutic target is the phosphatidyl inositol 3-kinase/AKT/mammalian target of rapamycin (PI3K/AKT/mTOR) pathway [5], [21], [22]. mTOR, an evolutionary conserved serine/threonine kinase is the catalytic subunit of two distinct complexes, mTORC1 and mTORC2, with different functions [23], [24], [25], [26], [27]. Two recent studies have elucidated the importance of targeting both complexes, as treatment of leukemic cells and patient-derived leukemic progenitors with ATP-competitive inhibitors of mTOR, such as OSI-027 and PP242, elicited strong anti-leukemic responses, even in the case of T315I-BCR-ABL-expressing cells [28], [29].

Sestrins are a family of stress-inducible proteins, consisting of Sestrin1 (SESN1), Sestrin2 (SESN2) and Sestrin3 (SESN3). These proteins share high homology with the bacterial AhpD protein, responsible for catalyzing the reduction of peroxiredoxins (Prdx), enzymes that metabolize peroxides [30]. The *Sesn1* and *Sesn2* genes are under transcriptional control of p53, while SESN3 is transcriptionally regulated by the AKT/FOXO axis, through FOXO1/FOXO3a-mediated gene expression [30], [31]. Recently, sestrins have been implicated in the regulation of mTORC1 signaling, as ectopic expression of any of the three sestrins results in inhibition of phosphorylation of mTORC1 pathway effectors [32]. Sestrin-mediated inhibition of mTORC1 occurs via AMPK-mediated activation of the TSC1/2 complex [32]. Through an unknown mechanism, sestrins interact with and allow for activation of AMPK, while also facilitating the interaction between AMPK and the TSC1/TSC2 complex [32], [33]. Moreover, abolishing either SESN1 or SESN2 expression reverses mTORC1 signaling suppression under basal and stress conditions [32]. The third family member, SESN3, also inhibits mTORC1 as evidenced by the findings that the FOXO1/3a-mediated transcriptional upregulation of SESN3 leads to activation of the AMPK/TSC1/2 axis, thereby inhibiting mTORC1 activity [34]. SESN3 also mediates ROS detoxification by FOXO as well as inhibition of cellular senescence [35].

The initial link between oncogenesis and sestrins arose from the studies of Ras expression, concluding that Ras suppresses the sestrin family of genes [36]. As mTOR signaling is an attractive drug target in a number of malignancies, upon elucidation of the sestrins-mTORC1 crosstalk, studies were conducted focusing on the link between sestrins and cancer via the mTORC1 and AMPK axis. Ablation of drosophila sestrin (dSESN) enhanced TORC1-stimulated hyperplastic growth [37], while overexpression of SESN1 or SESN2 suppressed hyperactive mTORC1-mediated malignant cell growth [32], [38].

In the present study, we examined the effects of BCR-ABL and mTOR inhibition on SESN3 expression. Our data demonstrate that treatment with first and second-generation TKIs induce expression of SESN3 levels in wild-type BCR-ABL expressing cells, while the third-generation TKI ponatinib induces SESN3 expression even in cells harboring the T315I-BCR-ABL mutant. SESN3 induction is mTOR-dependent, as treatment with the ATP-competitive mTORC1/2 inhibitor OSI-027 also results in SESN3 induction. Importantly, overexpression of SESN3 results in partial inhibition of mTORC1 and exhibits potent suppressive effects on leukemic cells expressing either WT-BCR-ABL or mutant TKI-resistant BCR-ABL, suggesting a mechanism for generation of antileukemic responses.

## Materials and Methods

### Cells and Reagents

The human Ph+ ALL cell lines BV173 and BV173R were kindly provided by Dr. Nicholas J. Donato (University of Michigan, Ann Arbor, MI) [28]. The Ba/F3 p210T315I mouse stable transfectants were kindly provided by Dr. Brian J. Druker (Howard Hughes Medical Institute and Oregon Health & Science University, Portland, OR) [28]. The U937 AML cell line was obtained from ATCC. The KT-1, BV173, BV173R, U937 and Ba/F3 cell lines were grown in RPMI 1640 medium supplemented with fetal bovine serum and gentamicin. Antibodies against the phosphorylated forms of AKT, ribosomal protein S6 and ERK and total forms of AKT, ribosomal protein S6 and ERK were purchased from Cell Signaling Technology, Inc. Antibodies against SESN2 and SESN3 were purchased from Proteintech. Antibodies against the phosphorylated form of PRAS40 were purchased from EMD Millipore. Antibodies against the total form of PRAS40 were purchased from Life Technologies. Rapamycin was purchased from Calbiochem/EMD. Imatinib mesylate and ponatinib were purchased from ChemieTek. OSI-027 was purchased from ChemieTek. In some initial studies the compound from OSI-pharmaceuticals was used. Nilotinib was from Chemie Tek. The AKT inhibitor MK-2206 was purchased from Santa Cruz Biotechnology, Inc. (catalog #sc-364537) The *SESN2* (catalog #SC128085) and *SESN3* (catalog #SC313706) human cDNA clones were obtained from OriGene.

### Cell lysis and Immunoblotting

Cell lysis and immunoblotting were performed as in previous studies [28], [39]. For such studies, the cells were treated with OSI-027 (5 µM), rapamycin (20 nM), imatinib mesylate (1 or 5 µM as indicated) or nilotinib (100 nM) for the indicated times, unless otherwise specified.

### Quantitative RT-PCR

Cells were treated with OSI-027, rapamycin, imatinib mesylate, nilotinib or ponatinib as indicated, and quantitative RT-PCR was carried out as in our previous studies [40]. Commercially available FAM-labeled probes and primers (Applied Biosystems) were used to determine SESN2 (Hs00230241_m1, Mm00460679_m1) and SESN3 (Hs00376220_m1, Mm01171504_m1) expression levels. GAPDH (Hs99999905_m1) or β-actin (Mm00607939_s1) primers were used for normalization as indicated.

### Statistical Analyses

Statistical significance was assessed using paired t-test analysis. p values are indicated in the relevant figures and/or the corresponding figure legends.

### Detection of ROS

Detection of ROS was conducted as previously described [41]. Briefly, cells were treated as noted. 30 minutes before end of treatment, half of the cells were collected by centrifugation at 4°C, 400 g for 5 minutes, washed with cold PBS and resuspended in 0.5 mL of cold PBS and kept on ice until the end the treatment (unstained control). The remaining cells were stained with 5 µM DCFDA dye (Invitrogen). At the end of treatment, the remaining cells were collected as described above. ROS measurements were obtained using the BD LSR Fortessa instrument at the RHLCCC Flow Cytometry facility.

#### Human leukemic hematopoietic progenitor cell assays

Clonogenic hematopoietic progenitor assays in methylcellulose to assess leukemic progenitor colony formation were performed as in previous studies [28], [39].

#### WST-1 assay

Cell proliferation was detected using the cell proliferation reagent WST-1 (Roche) [42]. Briefly, BV173R cells were transiently nucleofected with either empty vector (E.V.) or SESN2 or SESN3 expressing plasmids. 48 hours after transfection, cells were seeded in 96-well plates and proliferation was measured at the time points indicated. First, the cells were treated with WST-1 substrate for 2 hours, then, the cells were analyzed on a microplate reader (BioTek).

## Results and Discussion

**Figure 1:**
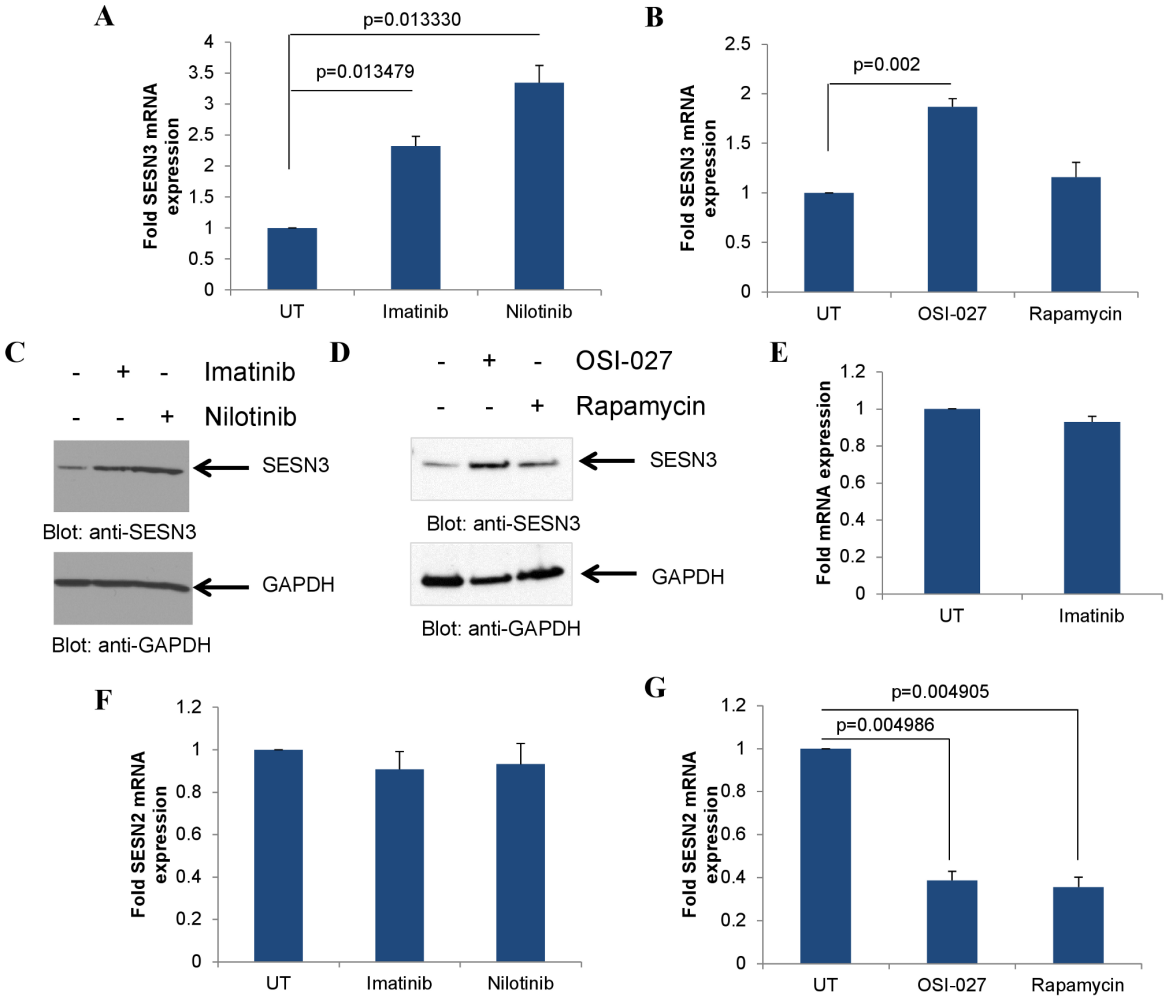
SESN2 and SESN3 expression upon BCR-ABL or mTOR inhibition. **A**. KT-1 cells were treated with either imatinib (1 µM) or nilotinib (100 nM) for 12 hours as indicated. RNA was extracted and expression of SESN3 mRNA was determined by quantitative RT-PCR, using GAPDH for normalization. Data are expressed as fold increase in the treated samples over untreated samples and represent means ± S.E. of 3 independent experiments. **B**. KT-1 cells were treated with either OSI-027 (5 µM) or rapamycin (20 nM) for 12 hours as indicated. RNA was extracted and expression of SESN3 mRNA was determined by quantitative RT-PCR, using GAPDH for normalization. Data are expressed as fold increase in the treated samples over untreated samples and represent means ± S.E. of 4 independent experiments. **C**. KT-1 cells were treated with imatinib (1 µM) or nilotinib (100 nM) for 24 hours. Total cell lysates were resolved by SDS-PAGE and immunoblotted with antibodies against SESN3 or GAPDH as indicated. **D**. KT-1 cells were treated with OSI-027 (5 µM) or rapamycin (20 nM) for 16 hours. Total cell lysates were resolved by SDS-PAGE and immunoblotted with antibodies against SESN3 or GAPDH as indicated. **E**. U937 cells were treated with imatinib (1 µM) for 12 hours. RNA was extracted and expression of SESN3 mRNA was determined by quantitative RT-PCR, using GAPDH for normalization. Data are expressed as fold increase in the treated samples over untreated samples and represent means ± S.E. of 3 independent experiments. **F**. KT-1 cells were treated with either imatinib (1 µM) or nilotinib (100 nM) for 12 hours. RNA was extracted and expression of SESN2 mRNA was determined by quantitative RT-PCR, using GAPDH for normalization. Data are expressed as fold increase in the treated samples over untreated samples and represent means ± S.E. of 3 independent experiments. **G**. KT-1 cells were treated with either OSI-027 (5 µM) or rapamycin (20 nM) for 12 hours. RNA was extracted and expression of SESN2 mRNA was determined by quantitative RT-PCR, using GAPDH for normalization. Data are expressed as fold increase in the treated samples over untreated samples and represent means ± S.E. of 3 independent experiments.

There have been previous studies implicating AKT in the regulation of SESN3 [34], while other work has implicated the AKT/mTOR pathway as an effector pathway for BCR-ABL-mediated responses [6], [7], [28], [29], [43], [44]. We sought to examine whether BCR-ABL exhibits regulatory effects on the expression of SESN3. Inhibition of BCR-ABL kinase activity using imatinib or nilotinib induced SESN3 expression in the WT-BCR-ABL-harboring cell line KT-1 (Fig. 1A). As BCR-ABL-expressing cells were found to have activated mTORC1 and mTORC2 complexes [28], [29], with the latter being responsible for AKT activation, we examined the effects of the ATP-competitive, dual mTORC1/2 inhibitor OSI-027 and the selective mTORC1 inhibitor rapamycin on the expression levels of SESN3. Treatment with OSI-027 potently induced SESN3 levels, while rapamycin had minimal effects (Fig. 1B). Consistent with the gene expression data, the BCR-ABL inhibitors imatinib and nilotinib as well as the mTORC1/2 inhibitor OSI-027 increased SESN3 protein levels (Fig. 1C and 1D respectively), while rapamycin had minimal effects (Fig. 1D). Moreover, imatinib treatment of the non-BCR-ABL expressing AML cell line U937 did not induce SESN3 expression (Fig. 1E), suggesting that the upregulation of SESN3 by imatinib is specific to BCR-ABL expressing cells. Interestingly, TKI treatment did not alter the gene expression of SESN2 (Fig. 1F), while on the contrary mTOR inhibition resulted in some decrease in SESN2 expression (Fig. 1G), indicating differential modulation of expression of different sestrin types in BCR-ABL transformed cells. As SESN3 has been found to be modulated by the AKT/FOXO pathway [34], [35], we examined the effects of the specific AKT inhibitor MK-2206, which inhibits AKT kinase activity (Fig. S1A). Treatment of KT-1 cells with the AKT inhibitor MK-2206 induced SESN3 expression, but no effects were observed on SESN2 expression (Fig. S1B).

**Figure 2:**
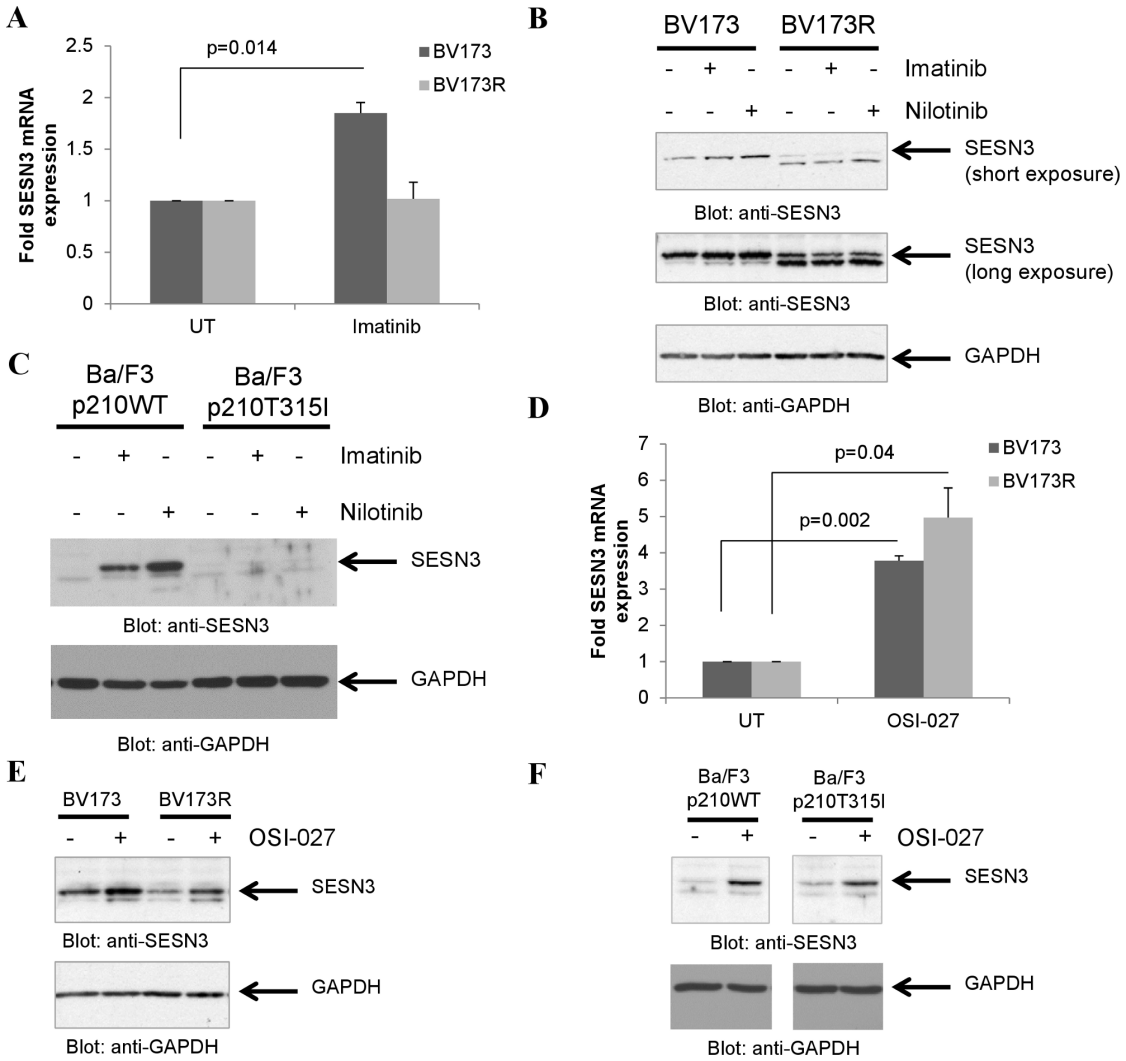
mTOR inhibition but not BCR-ABL inhibition upregulates SESN3 in T315I-BCR-ABL expressing cells. **A**. BV173 or BV173R cells were treated with imatinib mesylate (5 µM) for 12 hours. RNA was extracted and expression of SESN3 mRNA was determined by quantitative RT-PCR, using GAPDH for normalization. Data are expressed as fold increase in the treated samples over untreated samples and represent means ± S.E. of 3 independent experiments. **B**. BV173 or BV173R cells were treated with either imatinib (5 µM) or nilotinib (100 nM) for 16 hours. Total cell lysates were resolved by SDS-PAGE and immunoblotted with antibodies against SESN3 or GAPDH as indicated. **C**. Ba/F3 cells stably transfected with WT-BCR-ABL or T315I-BCR-ABL were treated with either imatinib (1 µM) or nilotinib (100 nM) for 16 hours. Total cell lysates were resolved by SDS-PAGE and immunoblotted with antibodies against SESN3 or GAPDH as indicated. **D**. BV173 and BV173R cells were treated with OSI-027 (5 µM) for 12 hours. RNA was extracted and expression of SESN3 mRNA was determined by quantitative RT-PCR, using GAPDH for normalization. Data are expressed as fold increase in the treated samples over untreated samples and represent means ± S.E. of 3 independent experiments. **E**. BV173 or BV173R cells were treated with OSI-027 (5 µM) for 16 hours. Total cell lysates were resolved by SDS-PAGE and immunoblotted with antibodies against SESN3 or GAPDH as indicated. **F**. Ba/F3 cells stably transfected with WT-BCR-ABL or T315I-BCR-ABL were treated with OSI-027 (5 µM) for 16 hours. Total cell lysates were resolved by SDS-PAGE and immunoblotted with antibodies against SESN3 or GAPDH as indicated.

To further determine whether the kinase activity of BCR-ABL is required for SESN3 expression, we examined the effects of TKIs on SESN3 mRNA expression in cells harboring WT-BCR-ABL as compared to cells harboring the TKI mutant T315I-BCR-ABL. Imatinib mesylate treatment induced SESN3 expression in the imatinib-sensitive Ph+ ALL cell line BV173 but failed to do so in the imatinib-resistant, T315I-BCR-ABL expressing [45] cell line BV173R (Fig. 2A). Additionally, both imatinib and nilotinib upregulated SESN3 protein levels in BV173, but not in BV173R cells (Fig. 2B). Similar results were seen when Ba/F3 p210WT-BCR-ABL and Ba/F3 p210-T315I-BCAR-ABL cell lines were used (Fig. 2C).

Effective targeting of both mTORC1 and mTORC2 complexes has been shown to overcome resistance to TKIs and dual mTORC1/2 inhibitors potently targeted cells expressing WT-BCR-BL as well as the T315I mutant of the oncoprotein [28], [29]. To determine whether catalytic mTOR inhibition results in SESN3 expression in T315I-BCR-ABL cells, the effects of the dual mTORC1/2 inhibitor OSI-027 on SESN3 expression were subsequently examined. OSI-027 treatment induced SESN3 mRNA expression (Fig. 2D) and increased SESN3 protein levels (Fig. 2E) in both the WT-BCR-ABL-expressing BV173 and in the T315I-BCR-ABL expressing BV173R cells. Similar results were obtained when Ba/F3 stable transfectants for WT-BCR-ABL or T315I-BCR-ABL were used (Fig. 2F).

**Figure 3:**
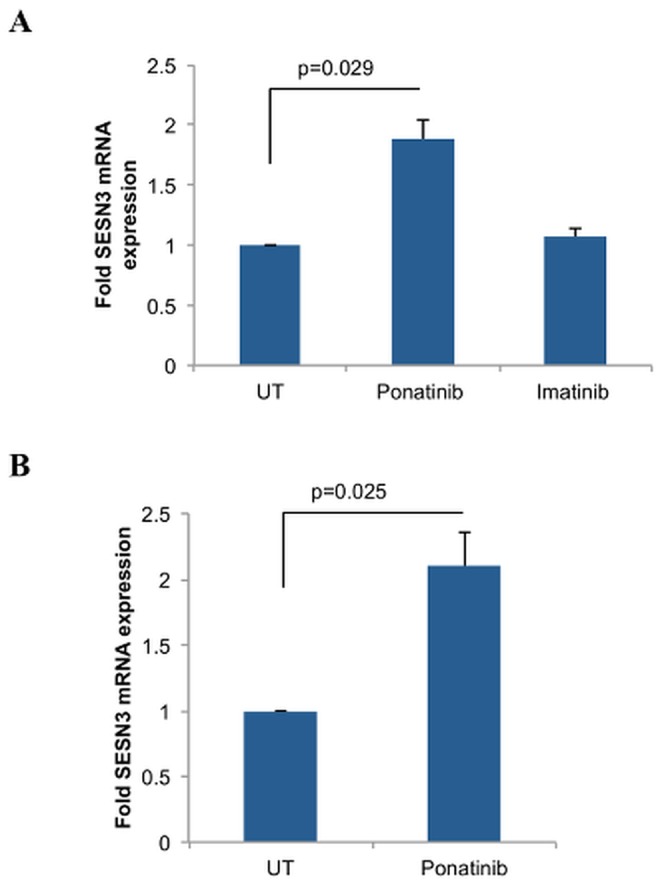
Induction of SESN3 mRNA expression by ponatinib in T315I-BCR-ABL expressing cells. **A**. Ba/F3 cells stably transfected with T315I-BCR-ABL were treated with ponatinib (10 nM) or imatinib (1 µM) for 12 hours as indicated. RNA was extracted and expression of SESN3 mRNA was determined by quantitative RT-PCR, using β-actin for normalization. Data are expressed as fold increase in the treated samples over untreated samples and represent means ± S.E. of 3 independent experiments. **B**. BV173R cells were treated with ponatinib (100 nM) for 12 hours. RNA was extracted and expression of SESN3 mRNA was determined by quantitative RT-PCR, using GAPDH for normalization. Data are expressed as fold increase in the treated samples over untreated samples and represent means ± S.E. of 4 independent experiments.

As the T315I mutation is resistant to first and second-generation TKIs, there has been an emerging focus on the effects of the novel third-generation TKI, ponatinib, which has efficacy against the T315I mutant form of BCR-ABL [15], [16]. Ponatinib treatment on T315I-BCR-ABL expressing Ba/F3 cells resulted in induction of SESN3 gene expression in the T315I-BCR-ABL expressing Ba/F3 cells, while imatinib treatment did not (Fig. 3A). Ponatinib also induced SESN3 expression in the Ph+ ALL T315I-BCR-ABL-expressing cell line BV173R (Fig. 3B), underscoring that inhibition of BCR-ABL by this novel third-generation TKI is sufficient for SESN3 induction.

**Figure 4:**
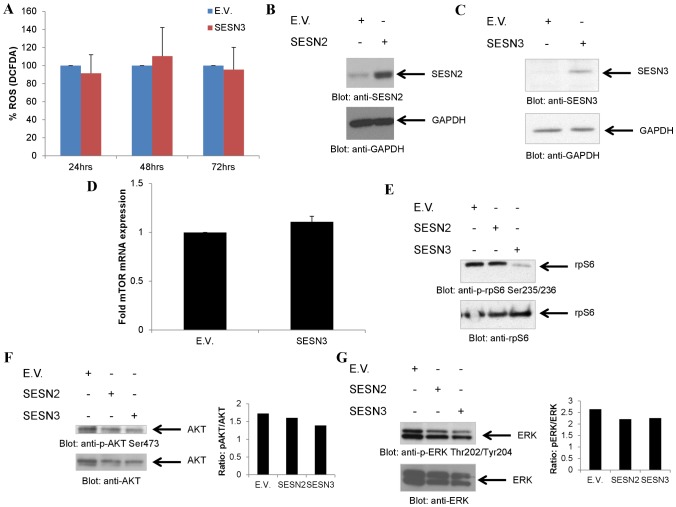
Differential effects of SESN3 and SESN2 overexpression on mTOR and MAPK signaling effectors. **A**. KT-1 cells were transiently nucleofected with either empty vector (E.V.) or SESN3 expressing plasmid and were analyzed for the presence of ROS by flow cytometry, following 30 minutes of staining with DCFDA at the time-points indicated. Data are as percent control empty vector for each time-point and represent means ± SE of 3 independent experiments. **B**. KT1 cells were transiently nucleofected with either empty vector (E.V.) or SESN2 expressing plasmid. Cells were lysed 48 hours post-nucleofection Total cell lysates were resolved by SDS-PAGE and immunoblotted with antibodies against SESN2 or GAPDH as indicated. **C**. KT1 cells were transiently nucleofected with either empty vector (E.V.) or SESN3 expressing plasmid. Cells were lysed 24 hours post-nucleofection Total cell lysates were resolved by SDS-PAGE and immunoblotted with antibodies against SESN3 or GAPDH as indicated. **D**. KT-1 cells were transiently nucleofected with either empty vector (E.V.) or SESN3 expressing plasmid. Expression of mTOR was quantified at 24 hours post-nucleofection either by quantitative RT-PCR. Data are expressed as fold increase in the Sesn3-nucleofected samples over E.V.-nucleofected samples normalized to GAPDH and represent means ± S.E. of 3 independent experiments. **E**. KT1 cells were transiently nucleofected with either empty vector (E.V.), SESN2 or SESN3 expressing plasmids. Cells were lysed 48 hours post-nucleofection and equal amounts of protein from cell lysates from the same experiment for each panel were resolved separately by SDS-PAGE and immunoblotted with the indicated antibodies. **F–G**. KT1 cells were transiently nucleofected with either empty vector (E.V.), SESN2 or SESN3 expressing plasmids. Cells were lysed 48 hours post-nucleofection and equal amounts of protein were resolved by SDS-PAGE and immunoblotted with the indicated antibodies. Blots were subsequently stripped and reprobed with the respective antibodies against the total form of the protein. Densitometry analysis of the representative blots is shown.

In subsequent experiments we sought to define the effects of sestrin overexpression in BCR-ABL transformed cells. There has previously been evidence for anti-tumorigenic effects of sestrins, primarily linked to modulation of ROS [36], while it is known that BCR-ABL expression results in ROS expression [21]. Thus, we examined the effects of SESN3 expression on ROS production in BCR-ABL transformed cells. Overexpression of SESN3 did not appear to decrease DCFDA staining, indicative of ROS, in this system (Fig. 4A). However, independent of ROS modulation, sestrins have been implicated in mTORC1 signaling regulation [30], [33]. We examined whether SESN2 or SESN3 have effects on S6 kinase (S6K) signaling by overexpressing either SESN2 (Fig. 4B) or SESN3 (Fig. 4C). Notably, SESN3 overexpression did not affect mTOR mRNA expression (Fig. 4D). SESN3, but not SESN2 overexpression reduced phosphorylation of rpS6 (Fig. 4E), reflecting inhibition of S6K activity. On the other hand, it did not have significant effects on phosphorylation of AKT on Ser473 (Fig. 4F), indicating that there were no effects on mTORC2 activity. Such effects appear to be specific, as SESN3 did not modulate the MEK/ERK pathway as phosphorylation of ERK was not altered (Fig. 4G). SESN2 overexpression did not induce changes in the phosphorylation of the proteins assessed, indicating that the effects on S6K inhibition are SESN3-specific (Fig. 4).

**Figure 5:**
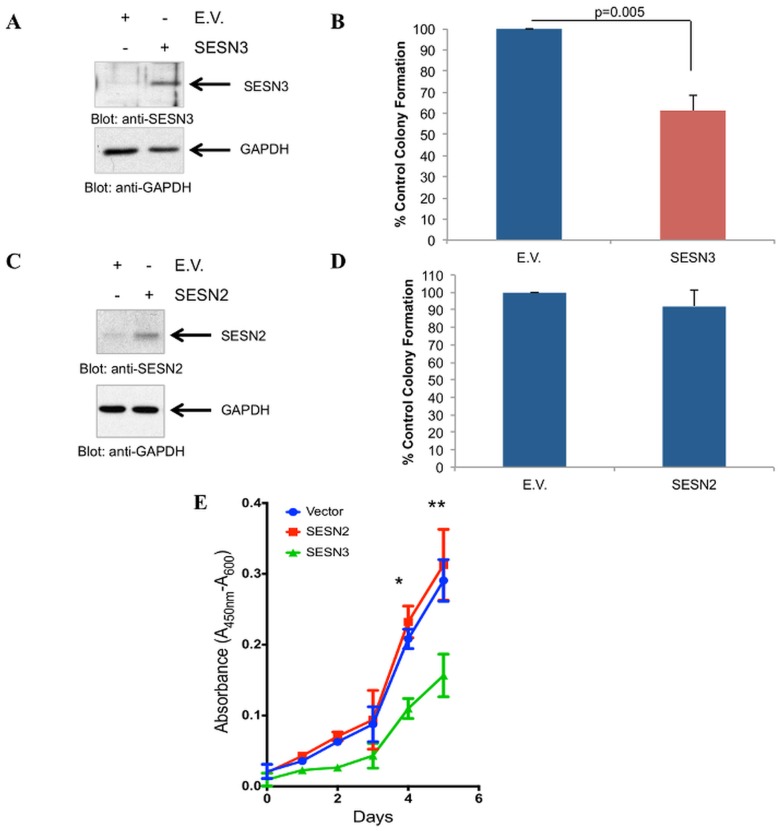
Inhibitory effects of SESN3 but not SESN2 on primitive BCR-ABL expressing leukemic progenitors. **A**. KT-1 cells were transiently nucleofected with either empty vector (E.V.) or a SESN3 expressing plasmid. Levels of SESN3 were quantified at 24 hours post-nucleofection by immunoblotting. **B**. KT-1 cells transiently nucleofected with either empty vector (E.V.) or SESN3 were plated in methylcellulose 24 hours post-nucleofection. Leukemic CFU-L colonies were allowed to develop in clonogenic assays in methylcellulose and scored on day 6. Data are expressed as percentage of control untreated colonies and represent means ± S.E. of 5 independent experiments. **C**. KT-1 cells were transiently nucleofected with either empty vector (E.V.) or SESN2 expressing plasmid. Levels of SESN2 were quantified at 24 hours post-nucleofection by immunoblotting. **D**. KT-1 cells transiently nucleofected with either empty vector (E.V.) or SESN2 expressing plasmid were incubated in clonogenic assays in methylcellulose. Leukemic CFU-L colonies were scored on day 6 and data are expressed as percentage of control untreated colonies and represent means ± S.E. of 4 independent experiments. **E**. BV173R cells were transiently nucleofected with either empty vector (E.V.) or SESN2 or SESN3 expressing plasmid. 48 hours post-transfection, equal number of cells were plated and allowed to proliferate for 120 hours. Proliferation was measured by WST-1 assay at the indicated times. Data are expressed as the absorbance at 450 nm and represent means ± S.E. from 3 independent experiments, * p = 0.0018 comparing SESN3 nucleofected cells vs. E.V. nucleofected cells on day 4, ** p = 0.0068 comparing SESN3 nucleofected cells vs. E.V. nucleofected on day 5.

Sestrins have been shown to have negative effects on mTORC1 signaling [34], [35], [37], [46], whose inhibition has been linked to anti-leukemic effects in BCR-ABL expressing cells [28], [29], [39]. To examine whether SESN3 is implicated in the generation of anti-leukemic responses, we overexpressed SESN3 in KT-1 cells and assessed the effects of such overexpression on leukemic CFU-L precursors, by clonogenic assays in methylcellulose. Overexpression of SESN3 in KT-1 cells (Fig. 5A) resulted in potent suppression of leukemic progenitor (CFU-L) colony formation as compared to controls (Fig. 5B). On the other hand, overexpression of SESN2 did not affect KT-1-derived leukemic progenitor colony formation (Fig. 5C and 5D), suggesting that SESN3, but not SESN2, is a selective mediator of antileukemic effects on BCR-ABL transformed leukemic precursors. Importantly, SESN3 overexpression (Fig. S2), led to a decrease in proliferation of the T315I-BCR-ABL harboring cell line BV173R (Fig. 5E).

While the sestrin family of proteins was first implicated in redox control [30], [31], [32], recent studies have been focusing on the role of sestrins in mTORC1 signaling. Particularly, sestrins appear to inhibit mTORC1 in an AMPK-dependent manner [33], [37], [46], [47] and previous studies have implicated SESN1 and SESN2 as negative regulators of mTORC1 activity and neoplastic cell proliferation [32]. However, the third member of the sestrin family of proteins, SESN3, has been studied to a lesser extent. There is some evidence this member of the family may be of importance in systems with high mTORC2 activity, as SESN3 is activated by the FOXO1/3a transcription factors, which are inhibited by the mTORC2/AKT signaling pathway [34], [35]. Interestingly, studies using *Drosophila* SESN (dSESN), showed that hyperactivation of TORC1 signaling induces dSESN [37]. An additional complexity in this mechanism is that TORC1 activation leads to ROS accumulation, resulting in JNK/FOXO signaling activation, which further induces dSESN expression [31], [37].

There has been prior evidence that CML and Ph+ ALL cells exhibit increased mTORC1 and mTORC2 activities, as it has been previously shown by us and others [28], [29], [39], while also harboring increased ROS levels compared to their non-transformed counterparts [21]. This led us to studies to determine whether sestrin expression is modulated by targeting BCR-ABL and/or inhibiting mTOR in Ph+ leukemic cells. To the best of our knowledge, our studies provide the first evidence that BCR-ABL inhibition by several different TKIs (imatinib mesylate, nilotinib, ponatinib) results in induction of SESN3 mRNA and protein expression. Likewise, inhibition of mTORC1/2 by OSI-027 also results in upregulation of SESN3. As the mTORC1/2 pathway is downstream of BCR-ABL [5], [48], [49], TKI-mediated increase of SESN3 likely results, at least in part, by mTORC1/2 inhibition and may be implicated in the generation of antileukemic responses as evidenced by the inhibitory effects of SESN3 on Ph+ leukemic precursors. Importantly, SESN3 induction by OSI-027 is observed in cells harboring the T315I-BCR-ABL mutant, further supporting our findings and underscoring the potential therapeutic relevance of mTOR targeting in BCR-ABL expressing leukemias, especially in the case of TKI resistance. Further confirmation of the role of BCR-ABL kinase activity in SESN3 suppression stems from studies using the third-generation TKI, ponatinib, which targets the T315I-BCR-ABL mutant, as ponatinib induces SESN3 upregulation in two different cell lines expressing the T315I-BCR-ABL oncoprotein. Future work should precisely define signals downstream of mTOR that control SESN3 expression, and also examine potential antileukemic effects of SESN3 on the 3 newly identified ponatinib-resistant BCR-ABL mutants L248R, T315V and F317R [17]. As our studies demonstrate that SESN3 promotes antileukemic responses, such future efforts may lead to the development of novel antileukemic agents that may selectively promote SESN3 expression and function and could be of value in the treatment of Ph+ leukemias alone or in combination with TKIs and/or mTOR inhibitors.

## Supporting Information

Figure S1 **Effects of AKT inhibition on SESN2 and SESN3 expression.** **A**. KT-1 cells were treated with MK-2206 (1 µM) or vehicle control for 12 hours. Equal amounts of protein were resolved by SDS-PAGE and immunoblotted with an antibody against the phosphorylated form of PRAS40 on Thr 246. Equal amounts of protein from lysates from the same experiment for each panel were analyzed separately by SDS-PAGE and immunoblotted with the indicated antibodies. Phosphorylation of PRAS40 on Thr246 was partially inhibited, consistent with upstream inhibition of AKT activity by the inhibitor. **B**. KT-1 cells were treated with MK-2206 (1 µM) for 12 hours. Total RNA was extracted and expression of SESN3 mRNA was determined by quantitative RT-PCR, using GAPDH for normalization. Data are expressed as fold increase in the treated samples over untreated samples and represent means ± S.E. of 3 independent experiments.
(TIF)

Figure S2 **Nucleofection of BV173R cells with sestrin expressing plasmids.** BV173R cells were transiently nucleofected with either empty vector (E.V.) or SESN2 or SESN3 expressing plasmids. Levels of SESN2 and SESN3 were quantified at 48 hours post-nucleofection by quantitative RT-PCR (in triplicate). Data from an experiment are expressed as fold increase in SESN mRNA expression from the SESN2-nucleofected or SESN3-nucleofected over E.V.-nucleofected samples, normalized to GAPDH, and represent means of triplicates ± S.D.
(TIF)
