# Supplementary figures and images for: Regulatory Effects of Sestrin 3 (SESN3) in BCR-ABL Expressing Cells
Source: PLoS One. 2013 Nov 18;8(11):e78780. doi: 10.1371/journal.pone.0078780 (PMC3832611; doi:10.1371/journal.pone.0078780)

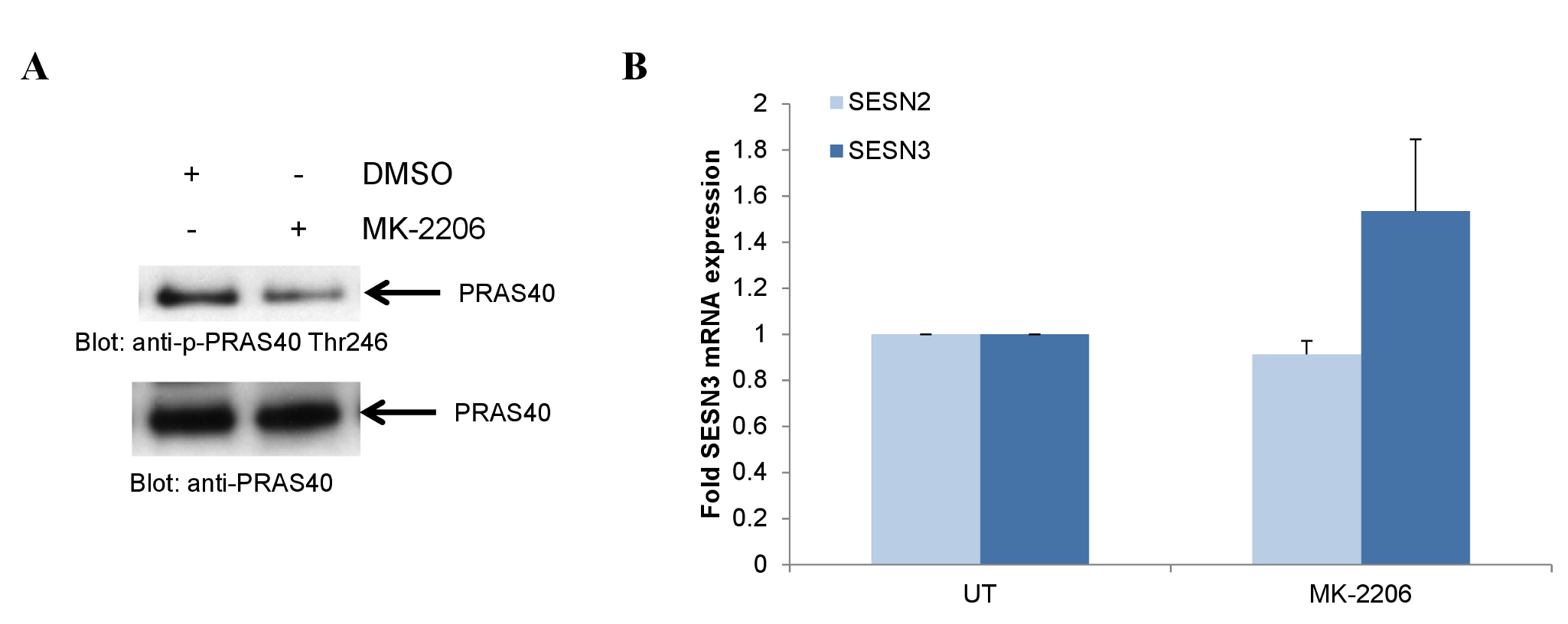

Supplement: Figure S1 — Effects of AKT inhibition on SESN2 and SESN3 expression. A. KT-1 cells were treated with MK-2206 (1 µM) or vehicle control for 12 hours. Equal amounts of protein were resolved by SDS-PAGE and immunoblotted with an antibody against the phosphorylated form of PRAS40 on Thr 246. Equal amounts of protein from lysates from the same experiment for each panel were analyzed separately by SDS-PAGE and immunoblotted with the indicated antibodies. Phosphorylation of PRAS40 on Thr246 was partially inhibited, consistent with upstream inhibition of AKT activity by the inhibitor. B. KT-1 cells were treated with MK-2206 (1 µM) for 12 hours. Total RNA was extracted and expression of SESN3 mRNA was determined by quantitative RT-PCR, using GAPDH for normalization. Data are expressed as fold increase in the treated samples over untreated samples and represent means ± S.E. of 3 independent experiments. (TIF) [file pone.0078780.s001.tif]
